# Pilot Sub-Study of the Effect of Hepatitis C Cure by Glecaprevir/Pibrentasvir on the Gut Microbiome of Patients with Chronic Hepatitis C Genotypes 1 to 6 in the Mythen Study

**DOI:** 10.3390/ph14090931

**Published:** 2021-09-16

**Authors:** Bahtiyar Yilmaz, Lisa Ruckstuhl, Beat Müllhaupt, Lorenzo Magenta, Melanie Harrer Kuster, Olivier Clerc, Ralph Torgler, Nasser Semmo

**Affiliations:** 1 Department for Biomedical Research, Inselspital, University of Bern, 3008 Bern, Switzerland; 2 AbbVie Schweiz AG, 6330 Cham, Switzerland; lisa.ruckstuhl@abbvie.com (L.R.); melanie.harrerkuster@abbvie.com (M.H.K.); ralph.torgler@abbvie.com (R.T.); 3 Department of Gastroenterology & Hepatology, University Hospital Zurich, 8091 Zurich, Switzerland; beat.muellhaupt@usz.ch; 4 Fondazione Epatocentro Ticino, 6900 Lugano, Switzerland; lorenzo.magenta@hin.ch; 5 Infectious Diseases Department, Hospital Pourtalès, 2000 Neuchâtel, Switzerland; olivier.clerc@rhne.ch; 6 Hepatology, Department for BioMedical Research, University of Bern, 3008 Bern, Switzerland

**Keywords:** HCV, gut microbiota, alpha-diversity, beta-diversity, direct-acting antivirals

## Abstract

In this small pilot sub-study, longitudinal gut microbiota composition changes, after successful treatment of hepatitis C virus (HCV) with the co-formulated glecaprevir/pibrentasvir (GLE/PIB), were analyzed before treatment (baseline) and 12 weeks post-treatment. Participating patients provided a fresh stool sample the week before their study visit, from which microbial DNA was extracted and sequenced for the 16S rRNA region in an Illumina MiSeq2 platform. Microbial and statistical analyses were conducted to determine the alpha-diversity (number of different taxa within a sample) and beta-diversity (number of overlapping taxa between samples). Stool samples from 58 patients were eligible for analysis. There were 27 patients with HCV genotype 1, 10 with genotype 2, 16 with genotype 3, and 5 with genotype 4. No statistically significant differences in gut microbiota diversity, species richness, or microbial community pattern were found at baseline and at post-treatment Week 12. Lack of statistically significant differences remained consistent in further analysis by demographic and baseline disease characteristics. Surprisingly, no statistically significant changes in alpha- and beta-diversity were seen in the microbiota after GLE/PIB treatment, though there was a trend toward less richness over time. Further investigation is needed into this unexpected outcome to better understand the role of HCV treatment and the gut microbiota.

## 1. Introduction

Taxonomic and functional changes to the composition of the gut microbiota have been implicated in multiple human diseases, ranging from gastroenterological disorders to neurological, respiratory, metabolic, hepatic, and cardiovascular illnesses. Significant changes in the gut microbiota have also been observed in patients positive for hepatitis C virus (HCV) compared with healthy controls and may be linked to developing comorbidities [1]. Co-evolved mutualism between the human immune system and the dense population of microorganisms present on our mucous membranes and body surfaces has promoted beneficial co-existence and interdependency over millions of years. The extended metabolic potential of biochemical pathways in microbes crucially contributes to human physiology, including digestive [2,3] and protective [4,5,6,7] functions (by out-competing the pathogens or via maturation of the host mucosal immune system) [8,9,10,11,12], catabolism of otherwise indigestible foodstuffs [13], provision of essential amino acids, synthesis of vitamins, completion of the bile salt cycle, and pre-systemic metabolism of drugs and toxins [14,15,16,17,18,19]. More than 70% of the total associated microorganisms in the human body live in the lower gastrointestinal tract [20].

Although the microbiota is relatively stable over time within individuals, changes can occur, and even at strain or sub-strain levels, it can be detrimental to host–microbial interactions with adverse overall health effects. When the composition of microbial consortia and their metabolic functions are altered, a host may experience loss of fitness, resulting in diseases such as inflammatory bowel disease [21], celiac disease [22], colorectal cancer [23], chronic inflammation [24], and metabolic diseases [25]. Conversely, many diseases themselves can alter the microbiota.

Changing biodiversity in the gut microbiota of patients with HCV is a current area of research interest. Patients who are HCV positive face a long list of potential health complications, including chronic liver disease, cirrhosis, and hepatocellular carcinoma [26]. Understanding the relationship of these comorbidities to gut microbiota could lead to important discoveries in treatment and management. One study has shown that chronic HCV infection is associated with statistically significantly less diversity in the gut microbiota compared with healthy controls [1]. As chronic HCV infections progress in severity from non-cirrhotic to cirrhotic, microbiota diversity continuously declines.

Worldwide, six different genotypes of the hepatitis C virus have been described as relevant for HCV infections in humans to date, with a varying global distribution [27]. Heidrich et al. observed a clear effect of HCV genotype on microbial diversity [1]. The study’s analysis revealed patterns in types of microbiotas in the healthy control, HCV cirrhotic, and HCV non-cirrhotic groups, indicating that the course of the disease could have a role in which organisms succeed in the altered microbial population.

In addition to the natural course of disease, it is also important to understand what effects HCV treatments, and thus viral clearance, may have on the gut microbiota. A number of available direct-acting antivirals can cure most HCV patients, with cure being defined as a sustained virologic response—that is, demonstrating lack of HCV RNA presence 12 to 24 weeks post-treatment [28]. Selecting a treatment for patients with HCV is nuanced, requiring considerations such as presence or absence of cirrhosis, treatment naivete, and which of the HCV genotypes a patient has [28]. A pangenotypic treatment regimen for HCV, co-formulated glecaprevir/pibrentasvir (GLE/PIB; NS3-4A protease inhibitor/NS5A inhibitor), was approved in 2017 in Switzerland (Maviret^®^ (GLE/PIB); AbbVie Inc., North Chicago, IL, USA) and can cure most patients with all six main genotypes 1–6 of HCV with a treatment duration of just 8 weeks [29]. A direct impact of glecaprevir/ pibrentasvir on the microbiota has not been investigated to date. In a recent study of GLE/PIB in a real-world setting [30], stool samples were collected as part of a secondary endpoint to provide data on the longitudinal change of the gut microbiota following HCV cure, a currently relatively unexplored area of research. In this small pilot sub-study, changes in the gut microbiota’s composition were analyzed before treatment (baseline) and in post-treatment Week 12 in patients routinely providing stool samples. 

## 2. Results

### 2.1. Population and Baseline Characteristics

Samples from 58 patients who consented to the gut microbiota analyses were eligible for analysis. Of these patients, 33 were men, five had cirrhosis, and 33 used illicit drugs (Table 1). The average age was 54.3 years, with seven patients aged ≥ 65 years. The mean body mass index was 24.0 kg/m^2^. There were 27 patients who had HCV genotype 1, 10 who had genotype 2, 16 who had genotype 3, and 5 who had genotype 4. No patients had genotypes 5 or 6. 

### 2.2. Gut Microbiota Analysis

**Figure 1:**
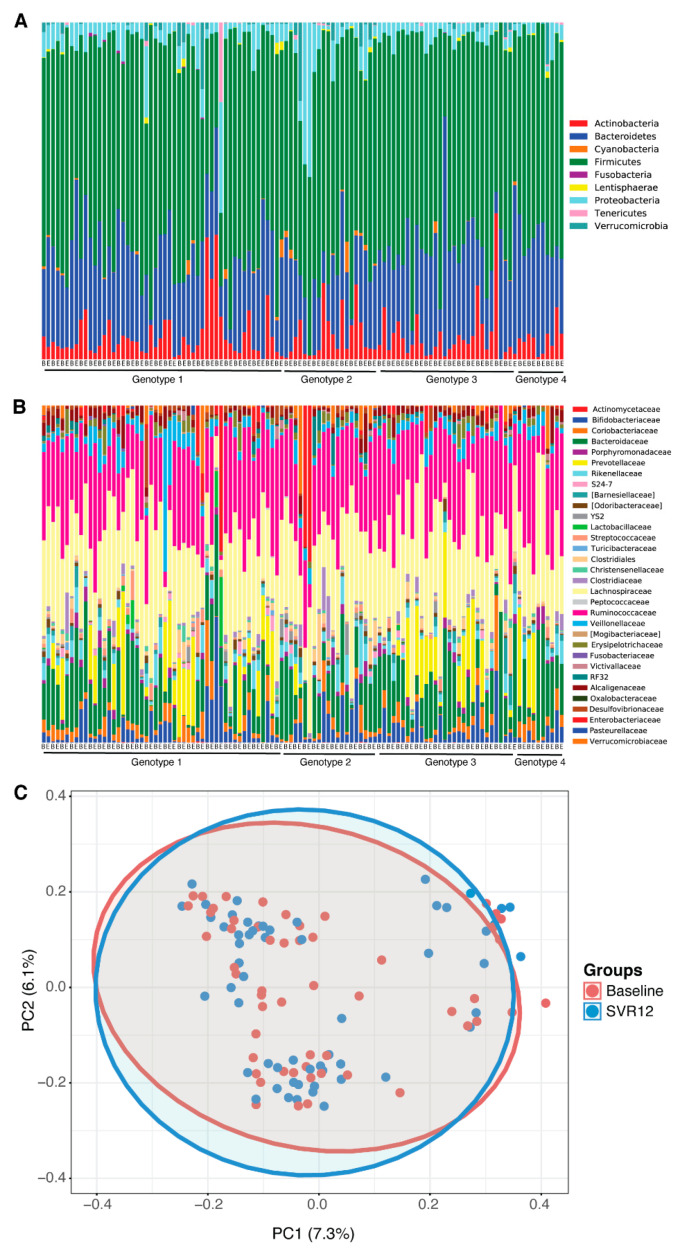
Microbial differences before treatment (baseline) vs. SVR12 groups. Taxonomy profile of patients at baseline and 12 weeks after the end of the treatment are shown (**A**) at phylum level and (**B**) at family level. (**C**) Microbial clustering is shown based on generalized UniFrac metrics using fecal DNA samples at baseline and SVR12. Non-parametric analysis of variance (Adonis) was used to test significant differences between groups on the PCoA plot, with a result of *p* > 0.05. The ellipses represent a 95% CI surrounding each disease group. B = baseline; E = end of treatment; PC = principal component; PCoA = principal coordinate analysis; SVR12 = sustained virologic response 12 weeks after the end of the treatment.

No statistically significant differences in the microbial community pattern were found before treatment (baseline) compared to post-treatment Week 12 in patients with HCV (Figure 1A–C). The microbiota profiles at phylum and family level were similar between baseline and sustained virological response 12 weeks after the end of the treatment (SVR12) (Figure 1A,B); when this was analyzed for beta-diversity profiles, the difference was not statistically significant (Adonis; *p* > 0.05).

**Figure 2:**
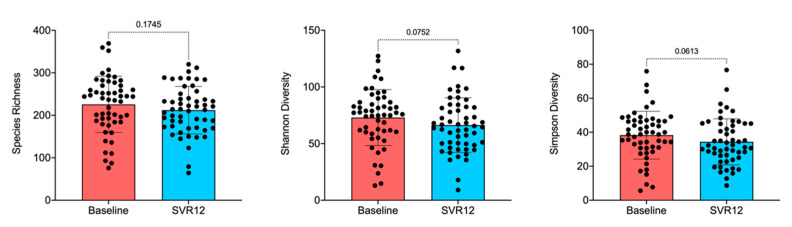
Overall species richness comparison of before treatment (baseline) and SVR12 groups. In this analysis, *p* < 0.05 was considered significant. Box-and-whisker plots display quartiles and range. SVR12 = sustained virologic response 12 weeks after the end of the treatment.

The box-and-whisker plot of species richness (Figure 2) indicates a decreasing trend in the number of taxa in the gut over time, although the difference was not statistically significant (Adonis; *p* > 0.05). This was still true when examined by virus genotype and timepoint (Figures S1 and S2, Supplementary Materials). Results calculated by the Shannon diversity were also confirmed using the Simpson diversity, which takes into account the number of species present as well as the abundance of each species. Samples were further analyzed for alpha-diversity by patient sex, illicit drug use, and virus genotype, but the decreases in microbiota diversity had no statistically significant differences before treatment compared to post-treatment Week 12 (data not shown). When analyzing alpha-diversity based on the virus genotype as measured by multivariate analysis by linear models R package [31], changes from baseline appeared notable for genotypes 1b, 3, and 4 and were thus examined by factors such as illicit drug use and patient sex (Figure S1, Supplementary Materials). Lack of statistically significant differences, however, remained consistent. 

**Figure 3:**
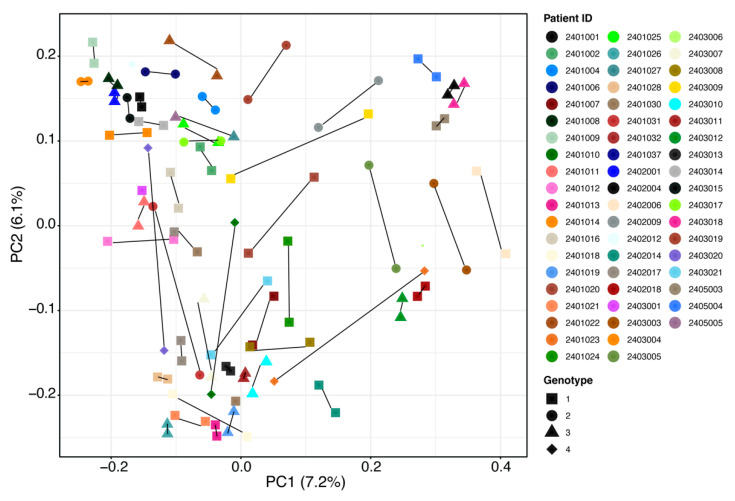
Overall beta-diversity differences before treatment (baseline) vs. SVR12 groups by patient and HCV genotype. Notable outliers with considerable changes are circled in red. Examples of minimal change are circled in blue for comparison. Non-parametric analysis of variance (Adonis) was used to test significant differences between groups on the PCoA (principal coordinate analysis) plot with a result of *p* > 0.05. SVR12 = sustained virologic response 12 weeks after the end of treatment.

Several outliers in the data (Figure 1C and Figure 2) were then further investigated by patients’ demographics and baseline disease characteristics to determine whether such changes were related to any characteristic of these patients. However, no statistically significant patterns for taxonomy profile (adjusted *p* value > 0.05) were seen for patient sex, age, illicit drug use, virus genotype, or drinking or smoking habits (Figure S3, Supplementary Materials). Results should be interpreted with caution owing to small sample sizes. Figure 3 shows individual patient changes in beta-diversity by virus genotype.

## 3. Discussion

At 12 weeks post-treatment with GLE/PIB in an observational real-world study, the gut microbiota of patients cured of HCV (i.e., patients demonstrating lack of HCV RNA presence 12 to 24 weeks post-treatment) had no statistically significant differences in diversity, richness, or microbial community pattern compared with baseline. The objective of this pilot sub-study was not to determine the effect of treatment on the microbiota, but rather to identify the effect that HCV cure may have on the microbiota. That the HCV cure had no significant effect on the microbiota was unexpected. Indeed, relative stability was instead seen before treatment compared to post-treatment Week 12 in patients cured of HCV. The question that then arises is why there were such small effects on the microbiota in most patients after treatment.

The lack of change is striking when considering the study factors that would be expected to cause variation but which had no apparent effect. Firstly, the sample size of 58 patients was relatively small, and, thus, any changes in outliers were likely to be amplified in the means. That no statistically significant changes occurred speaks to the robustness of the conclusion that no differences were found. Secondly, patient diet was not regulated for this real-world study setting. The consistency of the gut microbiota study findings, despite varied patient diets, further solidifies the results but leaves questions about what drives the general stability of the microbiota up to 12 weeks after HCV treatment. 

Several demographic features have been associated with negative effects to individual gut microbiota diversity, such as smoking, alcohol abuse, and illicit drug use [32]. No patterns were seen in the demographic or baseline characteristics of the outlying patients in whom large differences in alpha-diversity were recorded, and, therefore, no conclusions could be drawn as to why these individuals were different. 

There was a trend toward less richness by post-treatment Week 12, though this did not reach statistical significance. The relatively small change from pre-treatment to post-treatment Week 12 could reflect that the microbiota had not yet recovered to its healthy state during this follow-up period. On the other hand, without pre-infection data on these patients, it is not possible to determine if the status quo seen in this pilot sub-study was the healthy state itself, hence the stability in diversity. 

According to our findings, there is no direct effect of the virus on the microbiota. The differences in microbiota demonstrated by Heidrich et al. may instead explain an effect on the microbiota based on liver condition rather than from HCV itself [1]. Our pilot sub-study included only five patients who were cirrhotic, which does not allow a sub-analysis. Importantly, our pilot sub-study was intended to examine the effect of viral clearance on the microbiota, not the effect of the GLE/PIB treatment.

This study had several limitations. The small sample size was already noted, though minimal change in small numbers of patients instead supports the robustness of the findings. The study did not include many cirrhotic patients and, therefore, no definite conclusions could be drawn regarding this liver condition in relation to the gut microbiota post cure. The sequencing used in this study could only provide a good resolution of microbiota up to genus level. Shotgun metagenomics could be used to further test for any bacterial changes at the level of species, sub-species, and strains. A good starting point to investigate the microbial changes for clinical phenotype could be the prevalence of potentially pathogenic strains belonging to *Enterobacteriaceae* and *Streptococcaceae* with decreased presence of beneficial populations from the *Lachnospiraceae* family. 

Further research in this area should evaluate multiple timepoints after cure, perhaps up to 6 months, to ascertain longer term effects on the gut microbiota. It is not known how long the gut microbiota takes to recover following HCV treatment, but it is possible that it may follow the pattern of antibiotic treatment, in which recovery can take anywhere from several weeks to 2 to 6 months [33]. This should be investigated by long-term studies.

## 4. Materials and Methods

This was a prospective, multicenter, observational real-world effectiveness study in adult patients with chronic HCV genotypes 1 to 6 receiving the all-oral GLE/PIB regimen (full study design details in [30]). Patients could elect to participate in the routine stool sample collection for the gut microbiota analysis. This secondary endpoint was to evaluate changes in the composition of the gut microbiota (diversity, richness, microbial community pattern) from pre-treatment to post-treatment Week 12.

The study was conducted according to the guidelines of the Declaration of Helsinki and approved by the Ethics Committee Kantonale Ethikkommission Bern (protocol code P16-916 approved 27 February 2018). Informed consent was obtained from all patients involved in the study. A total of 109 patients were enrolled in the overall study. To be eligible to participate in the optional gut microbiota evaluation, patients must have routinely provided stool samples, not used antibiotics within 3 months before inclusion, and not had underlying diseases or behaviors that could influence the gut microbiota (e.g., inflammatory bowel disease, cancer, excessive chronic alcohol abuse, biotic diet), at the treating physician’s discretion.

Participating patients provided a fresh stool sample the week before their study visit. Samples were collected with diagnostic stool sample collection tubes and mixed with RNAlater (Ambion Inc., Austin, TX, USA). Afterward, they were stored at –20 °C. DNA extraction was performed using a QIAamp DNA Stool Mini Kit by following manufacturer’s instructions and the quantification of the dsDNA using PicoGreen^®^, and the integrity of a fraction of DNA isolations was checked by agarose gel electrophoresis. 16S rRNA next-generation amplicon sequencing was performed centrally (Microsynth AG, Balgach, Switzerland) on a MiSeq2 Illumina platform to analyze the gut microbiota [34]. 

The library preparation included sample quality control and Nextera two-step PCR amplification using primer set 341F_ill (CCTACGGGNGGCWGCAG) and 802R_ill (GACTACHVGGGTATCTAATCC) (V3/V4 region of 16S rDNA) and equimolar pooling after PCR product purification and amplicon concentration quantification. The demultiplexed paired-end MiSeq Illumina reads (2 × 300 bp) (Illumina, San Diego, CA, USA) were processed by using the QIIME2 pipeline as described [35] using custom analysis scripts for analysis on the UBELIX Linux cluster of the University of Bern (High Performance Computing, University of Bern, Bern, Switzerland) after sequence quality assessed using fastqc package [36]. Denoising, removal of chimeras, and dereplication were performed with the DADA2 pipeline implemented in QIIME2 (dada2 denoise paired). The software package Divisive Amplicon Denoising Algorithm 2 (DADA2) [37] was used to infer biological sequences from reads. Afterward, the extraction of the representative sequences using the “feature-table” and their classification by taxon using the “feature-classifier” were performed. The SILVA (version 132 [38]) database was customized following the instructions on the respective tutorials for QIIME2 for taxonomy assignment to OTUs. The taxonomy.gza, rep-seqs.qza, and rooted-tree.qza files generated in QIIME2 were called out in phyloseq pipeline in R [39,40]. Multivariate homogeneity of group dispersion was used to calculate the average distance of the groups and to test if the dispersion of any group was significantly different from the others. Multivariate analysis by linear models (MaAsLin) was used to find the taxa difference over time with potential associations of patient demographic and baseline disease characteristics (e.g., HCV genotype, sex, age) [31]. The clinical characteristic features were then also included as potential confounders in our multivariable regression model to test for the association between microbial species abundance versus treatment effect.

Taxa that were present in ≥30% of the samples and had >0.25% of total abundance were set as the cut-off values for further analysis as an effective threshold below which the analysis of spurious taxa can be prevented [41]. After Benjamini–Hochberg false discovery rate correction, adjusted *p*-value (<0.05) was considered significant.

Calculation of the α-diversity (species richness, Simpson diversity, and Shannon diversity) and β-diversity (Bray–Curtis genus-level community dissimilarities and generalized UniFrac-based PCoA), and statistical analysis of clustering using Mann–Whitney U tests for alpha-diversity and Adonis (PERMANOVA) for beta-diversity were performed in *phyloseq* in R [39,40]. Of note, alpha-diversity values were converted into effective numbers of species (also known as Hill numbers) by following the instruction on the papers based on Lou Jost’s proposition in 2006 [42,43,44].

## 5. Conclusions

To our knowledge, this pilot sub-study is the first to examine changes to the gut microbiota before treatment compared to post-treatment Week 12 in patients cured of HCV. Surprisingly, no statistically significant changes in alpha- and beta-diversity were seen in the microbiota after HCV cure with GLE/PIB treatment, though there was a trend toward less richness over time. Further investigation into this unexpected outcome is needed to better understand the role of HCV treatment and the gut microbiota.

## Figures and Tables

**Table 1:** Baseline characteristics.

Characteristics	Gut Microbiota Sub-Group *N* = 58
Mean ± SD age, years	54.3 ± 11.8
≥ 65, *n*	7
Mean BMI, kg/m^2^	24.0
≥ 30.0, clinically obese, *n*	4
Sex, *n*	
Men	33
Women	25
HCV genotype, *n*	
1 ^a^	27
2	10
3	16
4	5
Cirrhosis, *n*	5
Pre-treatment for HCV, *n*	6
Illicit drug use, *n*	33

BMI = body mass index; HCV = hepatitis C virus. ^a^ Includes all subtypes of HCV genotype 1.

## Data Availability

Data is contained within the article and supplementary materials. Further, raw sequencing data can be obtained upon request from Abbvie and Yilmaz.

## Supplementary Materials

The following are available online at https://www.mdpi.com/article/10.3390/ph14090931/s1. Figure S1: Overall alpha-diversity (species richness, Shannon diversity, and Simpson diversity) based on effective numbers of species by virus genotype; Figure S2: Overall species richness by virus genotype and timepoint. SVR12 = sustained virological response 12 weeks after the end of the treatment; Figure S3: Comparisons of alpha- and beta-Diversity for current alcohol and nicotine user or non-User groups.
