# Supplementary material for: Pilot Sub-Study of the Effect of Hepatitis C Cure by Glecaprevir/Pibrentasvir on the Gut Microbiome of Patients with Chronic Hepatitis C Genotypes 1 to 6 in the Mythen Study
Source: Pharmaceuticals (Basel). 2021 Sep 16;14(9):931. doi: 10.3390/ph14090931 (PMC8472424; doi:10.3390/ph14090931)
Supplement: Supplementary file 1 [file pharmaceuticals-14-00931-s001.zip › pharmaceuticals-1313009-SI.pdf]

pharmaceuticals

MDPI

Supplemental Data

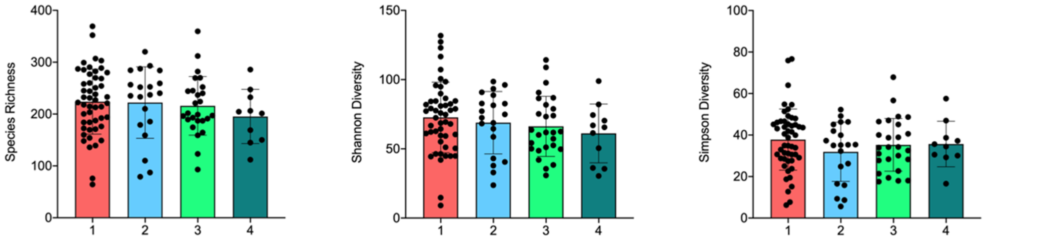

Figure S1. Overall Alpha-Diversity (Species Richness, Shannon, and Simpson Diversity) based on effective numbers of species by Virus Genotype.

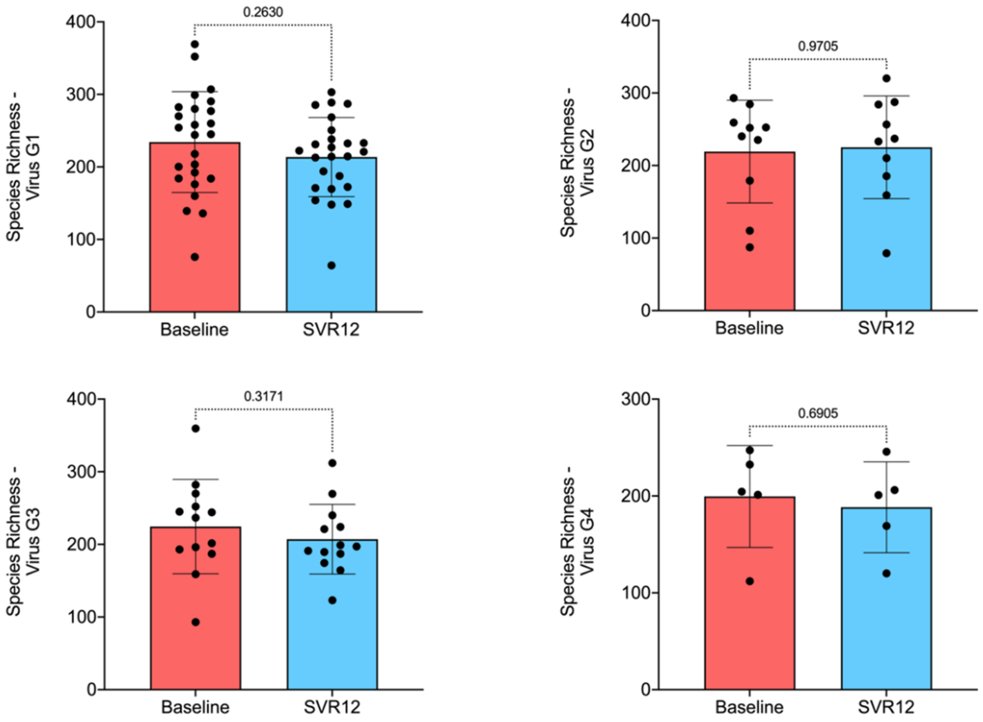

Figure S2. Overall Species Richness by Virus Genotype and Timepoint. SVR12 = sustained virological response 12 weeks after the end of the treatment.

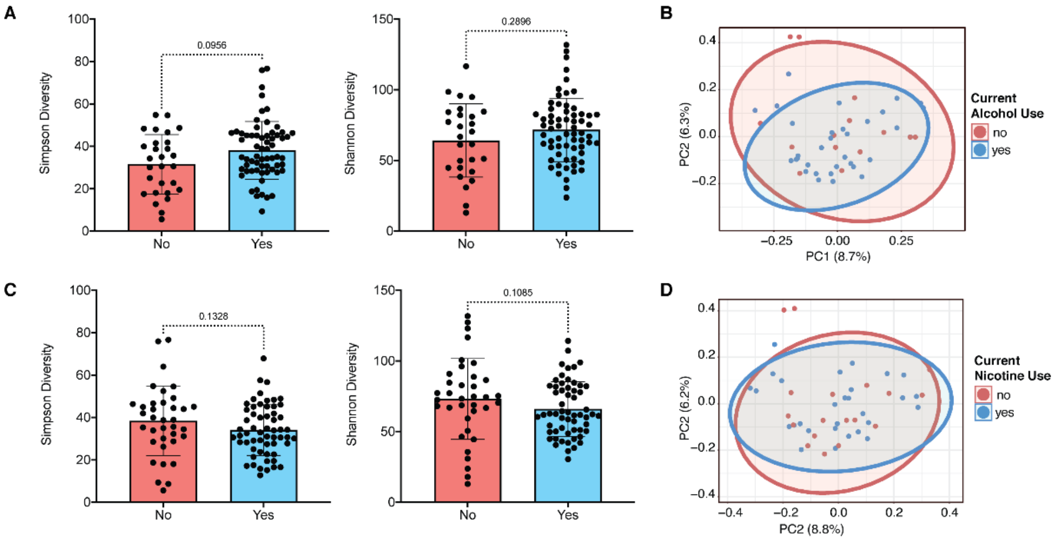

Figure S3. Comparisons of Alpha- and Beta-Diversity for Current Alcohol and Nicotine User or Non-User Groups. Alpha diversity (**Panel A**) using Simpson and Shannon diversities based on effective numbers of species and beta-diversity (**Panel B**) were analyzed by current alcohol use (yes) vs. no alcohol use (no). Alpha diversity (**Panel C**) using Simpson and Shannon diversities based on effective numbers of species and beta-diversity in (**Panel D**) were analyzed by current nicotine use (yes) vs. no nicotine use (no). The Mann-Whitney U-test was used to analyze significant differences between ranks ± SD. Box-and-whisker plots display quartiles and range. Non-parametric analysis of variance (Adonis) was used to test significant difference between groups on the PCoA plot, with a result of $p > 0.05$. The ellipses represent a 95% confidence interval surrounding each disease group. Results were not statistically significant in any panel. PCoA = principal coordinate analysis.
